# Treatment of Pipkin Type-III Femoral Head Fractures in Young and Middle-Aged Adults with Deep Circumflex Iliac Vascular Pedicled Iliac Bone Flap Transplantation

**DOI:** 10.12669/pjms.42.7.13398

**Published:** 2026-07

**Authors:** Junfeng Wan, Xiaojun Yu, Chao Hu, Lei Li

**Affiliations:** 1 Junfeng Wan, MD, Department of Orthopedics, Suining Central Hospital, Suining, Sichuan Province, China; 2 Xiaojun Yu, MD, Department of Orthopedics, Suining Central Hospital, Suining, Sichuan Province, China; 3 Chao Hu, MD, Department of Orthopedics, Suining Central Hospital, Suining, Sichuan Province, China; 4 Lei Li, MD, Department of Orthopedics, Suining Central Hospital, Suining, Sichuan Province, China

**Keywords:** Deep Circumflex Iliac Vascular Pedicled Iliac Bone Flap, Pipkin Type-III Femoral Head Fracture, Young and Middle-Aged Adults, Bone Transplantation

## Abstract

A retrospective analysis was conducted on the clinical data of two young and middle-aged male patients with Pipkin Type-III femoral head fractures admitted to our hospital from July 2022 to December 2022. Both patients were admitted due to hip pain and limited mobility caused by traffic accidents. Emergency hip dislocation reduction and femoral traction were performed first; after stabilization, the SP approach was used for bone flap transplantation plus internal fixation with proximal femoral locking plates and cannulated screws.Both patients had primary incision healing. No femoral head avascular necrosis or collapse occurred during two-year follow-up. At the final follow-up, the Harris hip scores were 92 and 94, indicating excellent functional recovery.

The combination of deep circumflex iliac vascular pedicled iliac bone flap transplantation and ORIF effectively promotes fracture healing and reduces femoral head necrosis, making it a feasible option for managing this fracture type in young and middle-aged adults.

## INTRODUCTION

Femoral head fracture is a rare, severe intra-articular injury from high-energy trauma-induced hip dislocation. Among Pipkin classifications, Type-III (combined femoral head-neck injury) has the worst prognosis, posing challenges for young and middle-aged patients with high hip function demands.^1^ Total hip arthroplasty (THA) is not ideal due to limited prosthesis life, so femoral head-preserving surgery is preferred.^1^,^2^

Open reduction and internal fixation (ORIF) are common initial treatment for this group, but severe femoral head blood supply damage leads to 42%-58% postoperative avascular necrosis and high nonunion rates, often requiring secondary THA. Insufficient revascularization (vessel damage + increased intra-articular pressure) is the core cause, with spontaneous repair difficult even after early reduction.^1^-^3^

Autologous vascularized bone transplantation addresses this: the deep circumflex iliac vascular pedicled iliac bone flap has stable anatomy and abundant blood supply, quickly establishing femoral head circulation post-transplantation and promoting bone repair.^2^,^4^ This study explored its efficacy combined with ORIF to reduce complications and provide a clinical option.

## CASE PRESENTATION

**Fig.1:**
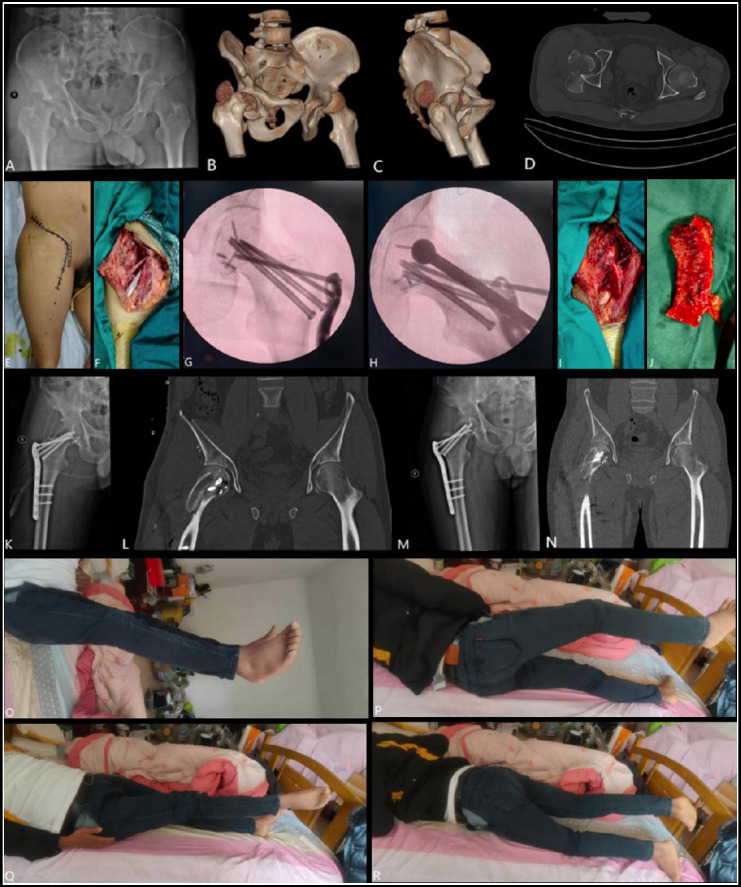
A thirty-seven-year-old male with right Pipkin Type-III fracture. (A) Preoperative X-ray. (B-D) Preoperative CT showing comminuted fractures. (E-F) Intraoperative SP approach. (G) Screw and locking plate fixation. (H) Femoral neck slotting and bone flap insertion. (I-J) Bone flap dissection showing bright red blood flow. (K) Postoperative X-ray. (L) Postoperative CT showing good reduction. (M) Two-year follow-up X-ray. (N) Two-year follow-up CT showing no necrosis and good healing. (O-R) Postoperative hip function showing good flexion, abduction, external rotation, and extension.

**Fig.2:**
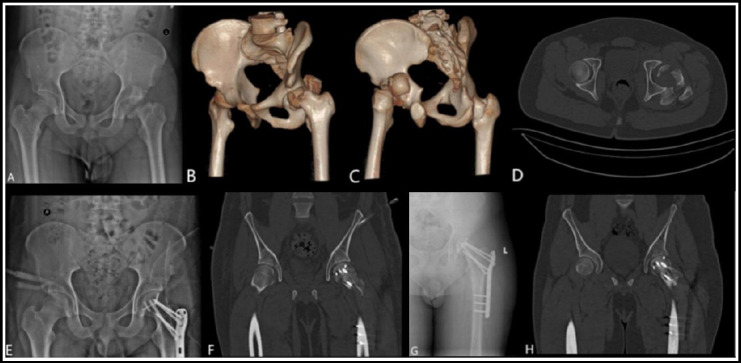
A nineteen-year-old male with left Pipkin Type-III fracture. Preoperative X-ray. (B-D) Preoperative CT showing comminuted fractures. (E) Postoperative X-ray. (F) Postoperative CT showing good reduction. (G) Two-year follow-up X-ray. (H) Two-year follow-up CT showing no necrosis and good healing.

Two male patients (thirty-seven and nineteen years old) were admitted after electric bike-car collisions, with hip pain and limited mobility. The older patient (right hip injury) was admitted two hours post-injury (Fig.1), the younger (left hip injury) four hours post-injury (Fig.2). Physical examination showed affected limb flexion/knee flexion/internal rotation deformity, hip swelling/tenderness, and normal distal circulation/sensation/movement. X-rays showed comminuted femoral head-neck fractures and hip dislocation.

Emergency hip reduction and femoral traction were done under general anesthesia; X-rays/CT confirmed fracture status post-stabilization. Hip-preserving surgery (SP approach) was performed: deep circumflex iliac vascular pedicled iliac bone flap transplantation plus fixation with proximal femoral locking plates and cannulated screws.

Key surgical steps: Elevate the affected hip; make an incision parallel to the iliac crest to anterior superior iliac spine, expose muscle intervals (protect lateral femoral cutaneous nerve); incise joint capsule, remove free bone fragments, create a femoral neck slot; reduce femoral head with “On Table” technique, fix with cannulated screws, then fix femoral head-neck with Kirschner wires temporarily; place proximal femoral locking plate and screws (confirmed by C-arm); harvest bone flap, pass vascular pedicle through iliopsoas tunnel, implant flap into the slot, fix with absorbable screws; recheck with C-arm, irrigate, suture. No postoperative surgical site infection was observed. Two-year follow-up showed no femoral head necrosis, bone graft fusion, and good lower limb function.

## DISCUSSION

Pipkin Type-III femoral head fracture is rare, involving both femoral head and ipsilateral femoral neck fractures. The principal therapeutic challenge stems from the dual insult to the femoral head’s blood supply: femoral neck fracture directly blocks main blood supply vessels like the medial femoral circumflex artery and increased intra-articular pressure from hip dislocation further aggravates ischemia, leading to a significantly higher postoperative avascular necrosis risk than other subtypes and poor prognosis.^1^,^3^ Due to its low incidence, most existing clinical studies are small-sample case series, and the optimal treatment approach is controversial. Wang Shanxi et al.^3^ conducted a retrospective analysis of twelve patients with Pipkin Type-III fractures who underwent ORIF, finding that 42% (5/12) developed femoral head necrosis (all requiring secondary THA) and 8% (1/12) converted to THA five months after initial surgery due to femoral neck fracture nonunion and internal fixation failure. Similar results were reported by Scolaro et al.^5^ (six out of seven patients who underwent initial ORIF developed femoral head necrosis) and Tonetti et al.^6^ (three out of four patients with Pipkin Type-III fractures developed femoral head necrosis after ORIF, all needing additional THA). Thus, more scholars advocate THA as the preferred treatment for Pipkin Type-III fractures.

However, femoral head fracture mainly affects young and middle-aged adults (average age at injury: 38.9 years),^4^ who have high demands for long-term hip joint function and activity. THA has obvious limitations in long-term efficacy for young patients: literature reports show that the twenty-year prosthesis survival rate is only 41%-66% for patients under thirty-five years old who undergo initial THA, and clinical evidence for initial THA in young patients with hip fractures is insufficient.^3^,^7^,^8^ Therefore, for young patients with Pipkin Type-III fractures, treatment strategies must balance “function preservation” and “complication prevention and control”. ORIF is still recommended as the preferred option to maximize preservation of the native hip joint structure, while THA is more suitable for elderly patients or cases with severe joint degeneration.^3^,^9^ The two patients in this study (aged nineteen and thirty-seven years) were in the high-activity stage; considering their long-term functional needs and prosthesis service life, THA was not suitable, so a treatment plan centered on “ORIF + revascularization” was finally determined.

To address insufficient femoral head blood supply post-fracture and reduce avascular necrosis risk, this study introduced deep circumflex iliac vascular pedicled iliac bone flap transplantation. This technique has advantages such as fixed anatomical position of the vascular pedicle, abundant blood perfusion, and simple harvesting. During pedicled transposition transplantation, it can directly establish blood supply connection with the ischemic area of the femoral head, providing direct osteogenic support through bone matrix and promoting membranous osteogenesis via growth factors from the vascular pedicle, thereby addressing the critical issue of revascularization in Pipkin Type-III fractures. During surgery, anatomical reduction of the fracture was first performed, followed by stable fixation with cannulated compression screws, thereby establishing a favorable environment for fracture healing and mitigating the risk of femoral head necrosis.

This treatment idea is supported by clinical studies: Lau et al.^2^ performed vascularized iliac bone transplantation on forty-two patients (fifty hips) with femoral head necrosis, and a seventeen-year follow-up showed that 56% (28/50) of the transplanted bone flaps survived long-term, verifying the technique’s long-term effectiveness. Pengfei Lei et al.^4^ studied eighteen young and middle-aged patients (nineteen hips) with femoral head necrosis, finding that 68.4% (13/19) had improved hip joint function twelve months after surgery, only 5.3% (1/19) required conversion to THA due to femoral head collapse, and the postoperative Harris hip score was significantly higher than before surgery (P<0.05), confirming the positive role of vascularized iliac bone flap transplantation in revascularization and functional recovery.

### Key surgical details to ensure efficacy include:

### Screw position and countersinking:

Screws in the femoral head area must be strictly countersunk to avoid protruding from the cartilage surface and causing joint wear; femoral neck screws should be placed as close to the inferomedial side as possible, combined with proximal femoral locking plate to achieve angular stability and enhance internal fixation strength.

### Protection of the vascular pedicle:

When dissecting the deep circumflex iliac vessel, part of the iliac muscle should be preserved; during bone flap harvesting, the integrity of the periosteum should be maintained as much as possible to avoid damaging the vascular pedicle; during bone flap transposition, the tunnel must be unobstructed to prevent vascular pedicle torsion, compression, or spasm and ensure stable blood supply.

In the present study, two-year follow-up showed no femoral head necrosis, bone graft fusion, and good lower limb function (Harris scores: 92 and 94).

## CONCLUSION

This study used “ORIF + deep circumflex iliac vascular pedicled iliac bone flap transplantation” to treat Pipkin Type-III femoral head fractures in young and middle-aged adults, providing a new and effective idea for clinical treatment amid the current lack of clear diagnosis and treatment guidelines. However, this study has limitations such as small sample size (only two cases) and relatively short follow-up time. In the future, expanding the sample size and prolonging the follow-up period will be essential to further validate the long-term efficacy and safety of this treatment regimen, providing more robust evidence for its clinical application.

### Authors’ Contributions:

**JFW:** Designed the study, did the manuscript writing and edited the final manuscript.

**XJY:** Did the operation. Critical Review

**CH:** Collected the clinical data followed up the patients.

**LL:** Takes the responsibility and is accountable for integrity of the study.

All authors have read and approved the final version of manuscript.
